# Comparison of cytological adequacy and pain scale score in ultrasound-guided fine-needle aspiration of solid thyroid nodules for liquid-based cytology with with 23- and 25-gauge needles: a single-center prospective study

**DOI:** 10.1038/s41598-019-43615-7

**Published:** 2019-05-07

**Authors:** Yoo Jin Lee, Dong Wook Kim, Gi Won Shin, Young Jin Heo, Jin Wook Baek, Hye Jung Choo, Young Jun Cho, Soo Jin Jung, Hye Jin Baek

**Affiliations:** 1 Department of Radiology, Busan Paik Hospital, Inje University College of Medicine, Busan, 47392 South Korea; 2 Department of Pathology, Busan Paik Hospital, Inje University College of Medicine, Busan, 47392 South Korea; 3 Department of Radiology, Gyeongsang National University Changwon Hospital, Gyeongsang National University School of Medicine, Changwon, 51476 South Korea

**Keywords:** Thyroid diseases, Thyroid cancer

## Abstract

In ultrasound (US)-guided fine-needle aspiration (FNA) of solid thyroid nodules (STN) using liquid-based cytology (LBC), the most appropriate needle size for LBC remains unclear. This study compared the cytological adequacy and complications associated with using 23- and 25-gauge needles in US-guided FNA of STNs using LBC. US-guided FNA was performed in consecutive patients by one radiologist to diagnose STNs ≥ 5 mm in the largest diameter. The one-sampling technique through a single needle puncture and multiple to-and-fro needle motions was used in each patient. The 23- and 25-guage needles were used consecutively each day. After FNA, the pain and complications experienced by each patient were investigated by a nurse, who was blinded to the information of needle gauge used. A cytopathologist retrospectively analyzed the cytological adequacy and cellularity of the cases. Of the 99 STNs, eight (8.1%) exhibited inadequate cytology (4 each with 23- and 25-gauge needles). The rate of cytological adequacy was not statistically different between the groups (*p* = 0.631). The mean pain scale values with 23- and 25-gauge needles were 2.1 ± 1.3 and 1.6 ± 1.3, respectively (*p* = 0.135). There were no significant complications in either group. In conclusion, both 23- and 25-gauge needles are useful in LBC because cytological adequacy and complications were not statistically different with both sizes of the needles.

## Introduction

Ultrasonography (US)-guided fine-needle aspiration (FNA) is a cost-effective modality used as the mainstay for initial evaluation of thyroid nodules^1^. For cytological diagnosis of US-guided FNA of thyroid nodules, conventional smears, which are prepared by coating the aspirated material evenly onto multiple microscope slides, have been used for a long time^1,2^. Liquid-based cytology (LBC) is rapidly replacing conventional smears since its introduction in 1996^3–6^. LBC employs advanced concentration techniques to yield a single slide that contains most, or all, of the FNA material^3,4^. Numerous factors can influence the cytological adequacy of US-guided FNA of thyroid nodules using LBC: the solidity or vascularity of the thyroid nodule, sampling technique used (aspiration and capillary acquisition) and number of samples taken (single versus multiple), number of needle passes, use of aspiration devices, and gauge of the needle used^1–5,7–11^. In clinical practice, needles between 21 to 27 gauge are usually selected according to the institution and physician^10,11^. While one previous study demonstrated that when solution-based samples are used, larger (21-gauge) needles provide more diagnostic specimen^7^, another study demonstrated that there was no significant difference between 21-gauge and 23-gauge needles^11^. Hence, the most appropriate needle size to be used for LBC remains unclear. This study aimed at comparing the cytological adequacy, pain scale, and other complications associated with using 23- and 25-gauge needles in US-guided FNA of thyroid nodules using LBC.

## Patients and Methods

### Patients

This prospective study was approved by the Busan Paik Hospital Institutional Review Board. All research was performed in accordance with relevant guidelines, and informed written consent was obtained from all patients before the US-guided FNA was performed. Between January and April 2018, one radiologist, with seven years of experience in US-guided FNA of thyroid nodules, performed US-guided FNA in 120 consecutive patients (94 female, 26 male; mean age, 51.2 ± 12.5 years; range, 24 to 77 years) to diagnose thyroid nodules that were ≥5 mm in the largest diameter (mean size, 15.2 ± 10.8 mm; range, 5.0 to 60.0 mm). Among these, purely calcified thyroid nodules (n = 5), thyroid cysts (n = 8), and partially cystic thyroid nodules (n = 8) were excluded. Ninety-nine patients (79 women, 20 men; mean age, 51.5 ± 12.6 years; range, 24 to 76 years) with solid thyroid nodules (defined as thyroid nodules with a solid component accounting for ≥90% of the total volume) were included.

### Ultrasound-guided fine-needle aspiration and pain scoring

**Figure 1:**
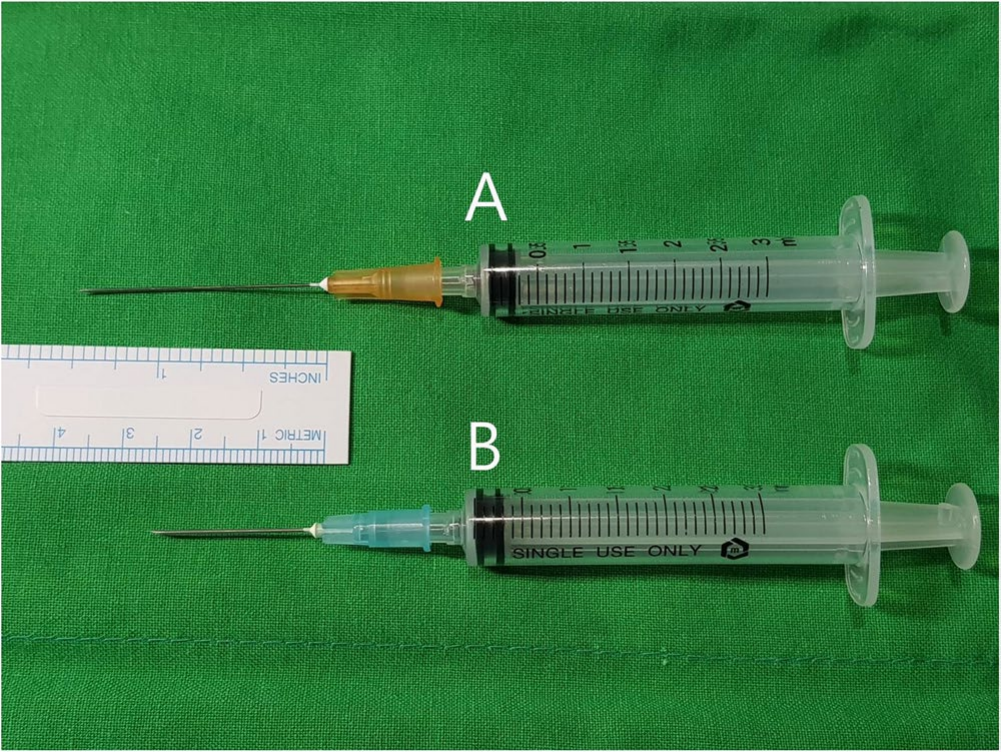
Fine-needle aspiration syringes attached to 25-gauge (**A**) and 23-gauge (**B**) needles.

One radiologist performed US-guided FNA in all patients under the guidance of a high-resolution US modality (iU 22; Philips Medical Systems, Bothell, WA, USA) equipped with a 5- to 12-MHz linear-array transducer. The operator used a conventional 23-gauge (2.5 cm in length) or a 25-gauge (3.8 cm in length) needle attached to an empty 3-mL plastic syringe (Fig. 1). For randomization of needle-size selection, the needle gauge was changed every other day. To obtain the specimen, the one-sampling technique including a single needle puncture and multiple to-and-fro needle motions was used, without applying an aspiration device nor local anesthesia^12^. To obtain adequate specimens, the mixed sampling technique (i.e., initial capillary sampling followed by progressive aspiration sampling depending on the amount of aspirate to be obtained) was used^12^. Aspirated material was collected in CytoLyt^TM^ solution (Hologic-Cytyc Co.) as follows: after separating the plastic syringe and needle, the contents within the syringe were injected into the LBC bottle, and then, the contents within the needle were flushed into the LBC bottle.

A post-procedure survey assessing pain and other complications after US-guided FNA had been routinely performed in our hospital. Immediately after completing the procedure, the patient was transferred to another room. Blinded to the needle size, a nurse asked the patient to grade the pain experienced during US-guided FNA using a pain scale with a numeric pain score ranging from 0 to 10 (0 represented “no pain”; 10 represented “the worst pain”). The nurse recorded the pain score mentioned by the patients.

### Liquid-based cytology and cytological analysis

After the CytoLyt^TM^ solution containing the aspirates was centrifuged, the obtained pellet was transferred to the PreservCyt^TM^ (Hologic-Cytyc Co.) solution and allowed to rest for 15 minutes. In each case, one Papanicolaou-stained slide was prepared by using ThinPrep 5000 (Hologic-Cytyc Co.).

All cytological slides were retrospectively analyzed by a cytopathologist with 16 years of experience in cytological analysis of thyroid FNA, with blinded to the data on the needle size. Specimen adequacy was determined according to the Bethesda System for Reporting Thyroid Cytopathology: six groups of ten well-visualized follicular cells was the minimal criteria^13,14^. Specimens that contained abundant colloid or numerous inflammatory cells were classified as adequate, even though six groups of follicular cells were not identified. On the contrary, specimens that only consisted of cystic contents were considered inadequate. To determine the cellularity, the numbers of cell clusters ≥ 10 well-visualized follicular cells were counted using a ×100 microscope in the highest cellular area. The cytopathologist classified the degree of cellularity as follows: high (more than 10 clusters), intermediate (5 to 10 clusters), and low (less than 5 clusters) cellularity. Cytomorphologic features were reviewed and categorized using the Bethesda classification.

### Statistical analysis

The data was tested for normal distribution using the Kolmogorov-Smirnov test. Age at the time of diagnosis, and the size of nodule were expressed as the mean ± SD. The cases were divided into two groups based on the size of needle used (23-gauge needle group versus 25-gauge needle group). Mean differences in age and size of nodule between the two groups were compared using the independent t-test. Group comparisons of categorical variables were performed using the χ^2^ test, and those for small cell values using the Fisher’s exact test. All statistical analyses were performed with a statistical software (SPSS, version 24.0, SPSS, Chicago, IL, USA), and P values < 0.05 were considered statistically significant.

### Ethics approval and consent to participate

The study design was approved by the appropriate ethics review boards (IRB 18-0135), and informed written consent was obtained from all patients before the US-guided FNA was performed.

### Consent for publication

Consent for publication was not obtained from the patient owing to patient anonymity and no patient details within the manuscript. All the authors have taken public responsibility for the manuscript content and publication.

## Results

The mean of the largest diameters of the 99 STNs was 14.4 ± 10.5 mm (range, 5.0 to 60.0 mm). The locations of the STNs included the right lobe (n = 50), left lobe (n = 48), and isthmus (n = 1). US-guided FNA was performed using 23-gauge needle for 49 (49.5%) STNs and 25-gauge needles for 50 (50.5%) STNs. The demographic characteristics of the two groups are presented in Table 1. The sizes of the STNs were higher in the 25-gauge group (18.0 ± 11.9 mm) than in the 23-gauge group (10.7 ± 7.4 mm) (*p* < 0.0001). However, there were no significant between-group differences in the patient age, gender, and location of STNs (*p* > 0.05).

**Table 1:** Comparison of demographic characteristics of the patient population according to the needle size in ultrasonography-guided fine-needle aspiration.

Items	23-gauge (n = 49)	25-gauge (n = 50)	*p* value
Age (yr)			0.551
mean	52.3 ± 11.6	50.7 ± 13.7	
range	25–76	24–75	
Gender			0.326
female	37 (46.8)	42 (53.2)	
male	12 (60)	8 (40)	
Location of STNs			0.269
right	28 (56)	22 (44)	
left	21 (43.8)	27 (56.3)	
isthmus	0	1 (100)	
Size of STNs (mm)			<0.0001
mean	10.7 ± 7.4	18.0 ± 11.9	
range	5–39.8	5–60	

Note. —Data are number of items, with percentage in parentheses. STN, solid thyroid nodule; US, ultrasonography.

Of the 99 STNs, eight (8.1%) showed inadequate cytology: six revealed no follicular cells, whereas two showed at least one follicular cluster with 20 to 60 follicular cells. The cytological adequacy and cellularity of the two groups are compared in Table 2. The rates of cytological adequacy in the 23- and 25-gauge needle groups were 91.8% (45/49) and 92% (46/50), respectively. This difference was not statistically significant (*p* = 0.631). The 25-gauge needle group showed a higher prevalence in the high cellularity class; the 23-gauge needle group showed a higher prevalence in the intermediate cellularity class; and both the groups had similar prevalence in the low cellularity class. The differences in the cytological cellularity between the groups were not statistically significant (*p* = 0.185). Among the 99 STNs, there were Bethesda category I (n = 8), II (n = 38), III (n = 25), IV (n = 6), V (n = 11), and VI (n = 11). Bethesda category II STNs were most common in both groups. There was no significant difference in the Bethesda classification between the two groups (*p* = 0.711).

**Table 2:** Comparison of clinicopathologic characteristics of the patient population according to the needle size in ultrasonography-guided fine-needle aspiration.

Items	23-gauge (n = 49)	25-gauge (n = 50)	*p* value
Cytological adequacy			0.631
adequate	45 (49.5)	46 (50.5)	
inadequate	4 (50)	4 (50)	
Cytological cellularity			0.185
low	27 (49.1)	28 (50.9)	
intermediate	12 (70.6)	5 (29.4)	
high	10 (37)	17 (63)	
Bethesda category			0.711
I	4 (50)	4 (50)	
II	20 (52.6)	18 (47.4)	
III	9 (36)	16 (64)	
IV	3 (50)	3 (50)	
V	7 (63.6)	4 (36.4)	
VI	6 (54.5)	5 (45.5)	
Pain scale			0.135
0	3 (23.1)	10 (76.9)	
1	12 (44.4)	15 (55.6)	
2	20 (54.1)	17 (45.9)	
3	7 (63.6)	4 (36.4)	
4	6 (75)	2 (25)	
5	0 (0)	1 (100)	
6	0 (0)	1 (100)	
7	1 (100)	0 (0)	
8	0 (0)	0 (0)	
9	0 (0)	0 (0)	
10	0 (0)	0 (0)	

Note. —Data are number of items, with percentage in parentheses.

The pain scale scores of the two groups are compared in Table 2. The mean pain scale values in the 23- and 25-gauge needle groups were 2.1 ± 1.3 (range, 0 to 7) and 1.6 ± 1.3 (range, 0 to 6), respectively. The 23-gauge needle group exhibited a higher score than the 25-gauge needle group. However, the difference in the scores between the two groups was not significant (*p* = 0.135). Scores of 1 or 2 were predominant, and no case reported a score more than 8. Intrathyroidal hemorrhage and other complications were not found in any case.

## Discussion

In US-guided FNA of thyroid nodules for conventional smears, thinner needles are associated with better cytological adequacy than larger needles^9,15^. However, for LBC, the relationship between the needle gauge used and the adequacy of the FNA specimen is still unclear. Although larger needles provide more specimen for diagnosis^7^, the difference in cytological adequacies between 21-gauge and 23-gauge needle groups is not significant^11^. Furthermore, the number of studies available is small. In the present study, we attempted to compare the difference in cytological adequacy of US-guided FNA of STNs for LBC between 23- and 25-gauge needles. There was no statistically significant difference. This result was similar to that of our previous study that compared 21-gauge and 23-gauge needles^11^. Moreover, the rates of cytological adequacy when using either 23- or 25-gauge needles were high (91.8% and 92%).Thus, we believe that the use of needles between 21- and 25-gauge does not affect the cytological adequacy of US-guided FNA of STNs for LBC.

In a previous study, samples collected using 21-gauge needles in US-guided FNA of thyroid nodules for LBC produced an average cellularity of 2.18, whereas those collected using 25-gauge needles produced an average cellularity of 1.92^7^. Although this difference was not statistically significant, the data trended toward suggesting that the use of 21-gauge needles for FNA increased the specimen cellularity (*p* = 0.08)^7^. Other investigators also reported that FNAs of thyroid nodules for LBC performed with 21-gauge needles led to more cellular specimens than those performed with 25-gauge needles^16^. However, the increased cellularity from the biopsies performed with the 21-gauge needles did not increase the sufficiency rate^16^. In our previous study, there was no significant difference in the cytological cellularity between 21- and 23-gauge needle groups. However, the 23-gauge needle group showed higher cellularity when compared with the 21-gauge needle group^11^. Likewise, the present study did not show a significant difference in the cytological cellularity between the 23- and 25-gauge needle groups, and the 25-gauge needle group showed higher cellularity when compared with the 23-gauge needle group. The reason for the disparity between our studies and others is not clear. We did not compare the difference in cytological cellularity between using 21- and 25-gauge needles.

A disadvantage of using larger needles is that they could result in more patient discomfort^11,15,16^. To the best of our knowledge, only one comparison study on US-guided FNA of STNs for LBC used the pain scale^11^. In that study using the one sampling technique, the mean pain score in the 23-gauge needle group (1.4 ± 1.1) was found to be lower than that in the 21-gauge needle group (1.8 ± 1.3), although this difference was not statistically significant^11^. In our study, although the pain score in the 23-gauge needle group (2.1 ± 1.3) was higher than that in the 25-gauge needle group (1.6 ± 1.3), the difference was not statistically significant. The reason for higher pain scores in the 23-gauge needle group may be associated with the smaller nodule sizes in this group than those in the 25-gauge needle group. Thus, we believe that when using the one sampling technique in US-guided FNA of STNs, patient pain does not differ in the use of needles between 21- and 25-gauge.

The following are the limitations of our study. First, there was a small sample size. Furthermore, the mean nodule size was different between two groups. This may be associated with a sampling bias. Second, 21 FNA cases with purely calcified thyroid nodules, thyroid cysts, or partially cystic thyroid nodules were excluded. Third, we did not investigate all the gauges of needles available (21 to 27-gauge). Fourth, the length of the 25-gauge needle was longer than that of the 23-gauge needle. This difference may have resulted in a bias. Finally, the histopathologic results of the 99 STNs were not investigated, as most of them were not confirmed by surgery.

In conclusion, the study results demonstrated that no significant differences were found in the cytological adequacy, cellularity, and pain scores between 23- and 25-gauge needles in US-guided FNA of STNs using LBC. Thus, we believe that both 23- and 25-gauge needles can be used in US-guided FNA of STNs using LBC.

## Data Availability

The datasets used and/or analyzed during the current study are available from the corresponding author on reasonable request.
